# A Comparative Analysis of the Effects of Cardioversion and Ablation on Anxiety, Sleep, and Quality of Life in Patients Diagnosed With Atrial Fibrillation

**DOI:** 10.1002/clc.70388

**Published:** 2026-06-23

**Authors:** Besey Oren, Busra Zehra Buyukkilic, Kubra Soydas, Ekin Oren

**Affiliations:** ^1^ Department of Internal Medicine Nursing, Hamidiye Faculty of Nursing University of Health Sciences Istanbul Turkey; ^2^ Dr. Siyami Ersek Thoracic and Cardiovascular Surgery Training and Research Hospital University of Health Sciences Istanbul Turkey; ^3^ Ruprecht‐Karls‐University of Heidelberg, Baden‐Wurttemberg, Faculty of Biosciences Heidelberg Germany

**Keywords:** anxiety, atrial fibrillation, cardioversion, catheter ablation, quality of life, sleep‐wake disorders

## Abstract

**Background and Aims:**

This study aims to evaluate changes in quality of life, anxiety levels, and sleep quality before and after elective electrical cardioversion or ablation in patients diagnosed with chronic atrial fibrillation (AF).

**Methods:**

This is a single‐center, quasi‐experimental study with a two‐group, pretest−posttest design conducted between January and December 2024 in a training and research hospital in Istanbul. A total of 57 patients who underwent either cardioversion (*n* = 30) or ablation (*n* = 27) were included. Data were collected using the AFEQT Quality of Life Questionnaire, Beck Anxiety Inventory, and Pittsburgh Sleep Quality Index, and analyzed with R software. Statistical analyses included Student's *t*‐test, Pearson's Chi‐square, Fisher's exact test, paired two‐sample *t*‐test, and Welch's unequal variance *t*‐test. A *p* value of < 0.05 was considered significant.

**Results:**

Post‐treatment quality of life significantly improved in both groups (AFEQT: ablation +21, cardioversion +11 points). Anxiety levels decreased in the ablation group (–4.7 points), while sleep quality showed no significant change (*p* = 0.092). In the cardioversion group, significant improvements were observed in both anxiety and sleep quality (BAI –8.63; PSQI –1.10; *p* < 0.001). Regression analyses showed significant correlations between quality of life, anxiety, and sleep both before and after treatment.

**Conclusion:**

Improvements in quality of life were observed following both ablation and cardioversion procedures in patients with AF. Greater reductions in anxiety and sleep disturbance scores were observed in the cardioversion group compared with the ablation group.

## Graphical abstract

In patients with atrial fibrillation, both catheter ablation and electrical cardioversion improved quality of life. Cardioversion was associated with greater short‐term reductions in anxiety and sleep disturbances, highlighting the importance of considering patient‐reported outcomes alongside rhythm‐control strategies.

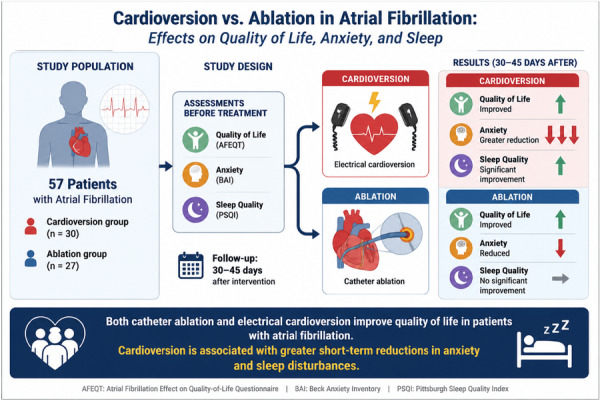

## 1 Introduction

Atrial fibrillation (AF) is the most common type of arrhythmia and is associated with heart failure, cerebrovascular events, dementia, and sudden cardiac death, making it a significant cause of morbidity and mortality [1]. The incidence of AF and its burden on healthcare systems worldwide are steadily increasing. Although the underlying mechanisms of AF remain not fully understood, it is known to be associated with various factors such as structural heart disease, hypertension, diabetes, and obesity [2].

Electrical cardioversion is a rapid, effective, and safe rhythm‐control strategy widely used for restoring sinus rhythm in patients with AF [3]. This procedure is typically performed electively in a hospital setting, requiring at least 1 day of hospitalization. The 2020 ESC and EACTS guidelines recommend electrical cardioversion as a Class I‐B treatment for hemodynamically unstable patients with AF [4]. Despite its widespread use and guideline recommendations, few contemporary clinical studies have investigated outcomes following cardioversion [5].

Catheter ablation is a treatment alternative for patients—particularly those with paroxysmal AF—who do not respond to pharmacological therapies [6]. The 2023 ACC/AHA/ACCP/HRS guideline recommends ablation at a Class I‐A level for symptomatic AF patients who are unresponsive to, intolerant of, or prefer not to take antiarrhythmic drugs [7]. The primary goal of ablation is the expected improvement in AF‐related symptoms and health‐related quality of life [6].

It is widely acknowledged that patients with AF experience a reduction in health‐related quality of life [8]. This decline is largely attributed to AF‐related symptoms, which also contribute to increased anxiety. Patients often reduce their social, physical, and occupational activities due to fear of complications, medication side effects, financial burden, and psychological impact of being labeled a “heart patient.” Several studies have shown associations between anxiety, depression, perceived stress, uncertainty, and reduced quality of life in patients with AF [9, 10]. In a 2019 study [11], also found that AF‐related anxiety, symptom frequency, and severity were linked to poorer quality of life.

Sleep disturbances, such as nocturnal intermittent tissue hypoxemia and heightened sympathetic activity, are known contributors to arrhythmias. The Sleep Heart Health Study reported a fourfold increase in AF prevalence in patients with an Apnea‐Hypopnea Index (AHI) of 30 or higher compared to those without sleep disorders, independent of other risk factors [12]. Sleep disorders are thus considered contributing factors in the development of AF [13]. A meta‐analysis by Zhang et al. [14] also confirmed a correlation between sleep disturbances and increased AF risk.

Although cardioversion and catheter ablation differ in terms of clinical indications, invasiveness, and patient selection criteria, both interventions are commonly used rhythm‐control strategies in patients with symptomatic AF. Since both procedures aim to restore or maintain sinus rhythm, evaluating their associations with patient‐reported outcomes such as quality of life, anxiety, and sleep quality may provide clinically meaningful insights for patient‐centered care and treatment planning. However, studies examining whether these two procedures differ in terms of sleep quality, quality of life, and anxiety are limited. Therefore, considering that the results of this study could guide care practices, these two treatment groups were compared.

## 2 Methods

### 2.1 Objectives of the Study

The findings from this study are expected to contribute to the development of patient‐centered strategies by identifying changes in quality of life, anxiety levels, and sleep quality before and after cardioversion or ablation procedures. This study evaluated changes in quality of life, anxiety levels, and sleep quality before and after cardioversion or catheter ablation procedures in patients diagnosed with AF.

### 2.2 Study Design

This is a single‐center, quasi‐experimental study with a two‐group, pretest‐posttest design. The sample included patients hospitalized with a diagnosis of AF in the cardiology department of a Training and Research Hospital under the Istanbul Provincial Health Directorate, who were scheduled for cardioversion or ablation and met the inclusion criteria.

### 2.3 Study Setting and Characteristics

The study was conducted between January and December 2024 in the cardiology department of a Training and Research Hospital under the Istanbul Provincial Health Directorate. This hospital was selected due to its specialized cardiology unit and frequent use of interventional procedures.

### 2.4 Study Sample

The study sample consisted of all eligible patients diagnosed with AF, hospitalized in the cardiology unit of the above‐mentioned hospital between January and December 2024, and scheduled for cardioversion or ablation by their physician.

#### 2.4.1 Inclusion Criteria

- Diagnosed with AF,
- Undergoing electrical cardioversion or ablation,
- Able to speak and understand Turkish,
- Literate,
- Voluntarily agreed to participate in the study,
- Adults aged 18 and above.

#### 2.4.2 Exclusion Criteria

- Diagnosis of dementia, Alzheimer's disease, or other cognitive impairments,
- Diagnosis of any psychiatric disorder,
- Use of sleep medications.

### 2.5 Sample Size Calculation

To determine the required sample size, a power analysis was conducted using the G*Power software (v3.1.9.7). Based on similar studies, the analysis was conducted at a 95% confidence level (*α* = 0.05) and 80% statistical power (1−*β* = 0.80), resulting in a minimum of 14 and a maximum of 36 participants. The average of these values suggested 25 participants per group, but to account for potential data loss and withdrawals, the sample size was increased by 20%, aiming for 30 participants per group (total *n* = 60). Due to one dropout in each group, the study was completed with 58 participants. All eligible patients hospitalized during the specified dates and undergoing the designated procedures were included.

### 2.6 Data Collection Tools

Data were collected using the Patient Information Form, Atrial Fibrillation Effect on Quality‐of‐Life (AFEQT) Questionnaire, Beck Anxiety Inventory (BAI), and Pittsburgh Sleep Quality Index (PSQI).

#### 2.6.1 Patient Information Form

Developed by the researchers based on the literature, this form collected data on age, gender, height, weight, education level, economic status, marital status, presence of chronic illnesses, medication use, emotional state on the day of the procedure, and adverse events or AF recurrence within the first 4 weeks post‐procedure. It also included questions on smoking habits and sleep quality.

#### 2.6.2 AFEQT Questionnaire

Designed to assess the impact of AF on patients' health‐related quality of life and potential treatment‐related changes. The Turkish validity and reliability study was conducted by Güneş and Boyraz [15], with a Cronbach's *⍺* of 0.91. Items are scored on a 7‐point Likert scale. Questions 19 and 20 assess treatment satisfaction and are excluded from the final score. Higher scores indicate a better quality of life.

#### 2.6.3 BAI

Developed by Beck et al. (1988), the Turkish version was validated by Avcı and İnceer [16], with a Cronbach's *⍺* of 0.94. It includes 21 items rated on a 4‐point Likert scale (0–3). Total scores range from 0 to 63, with higher scores indicating greater anxiety.

#### 2.6.4 PSQI

Developed by Buysse et al. (1989) and validated for Turkish use by Ağargün et al. [17], with a Cronbach's *⍺* of 0.804. The PSQI includes 24 items, 19 of which are self‐rated. Seven components are scored (0–3) and summed for a global score ranging from 0 to 21. Scores above 5 indicate poor sleep quality.

### 2.7 Data Collection and Ethical Considerations

Data were collected face‐to‐face by the researcher between January and December 2024. Eligible patients were informed about the study and provided both verbal and written consent. Pretest data were collected before the procedures, and posttest data were collected 30–45 days later during follow‐up visits or via telephone if necessary. Ethical approval was obtained (Meeting Date: 01.12.2023, Decision No: 20/17), and institutional permission was granted. The study adhered to the principles of the Declaration of Helsinki.

Major antiarrhythmic and cardiovascular medication regimens were routinely maintained during the short follow‐up period unless clinically indicated changes were required by the treating cardiologist. No major medication modifications that could substantially influence anxiety or sleep outcomes were recorded during follow‐up.

### 2.8 Statistical Analysis

Continuous variables (e.g., age, body mass index [BMI]) were summarized using means, standard deviations, and ranges. Differences between the ablation and cardioversion groups were assessed using Student's *t*‐test. Categorical variables (e.g., gender, marital status, education level, employment, smoking, chronic illnesses, medication use, stroke history) were analyzed using frequency distributions, Pearson's chi‐square test, or Fisher's exact test when expected cell counts were below 5. All statistical tests were two‐tailed, with a significance level set at *p* < 0.05. Analyses were conducted using R software (version 4.x).

## 3 Results

The study included 27 patients in the ablation group and 30 patients in the cardioversion group. No significant differences were found between the groups in terms of age (59.9 ± 13.4 vs. 61.2 ± 8.9 years; *p* = 0.668) or BMI (27.6 ± 4.8 vs. 25.5 ± 4.5 kg/m^2^; *p* = 0.101). Similarly, there were no statistically significant differences in categorical variables such as gender, marital status, education level, employment status, smoking, chronic illnesses (hypertension, diabetes, COPD, asthma, heart failure, arrhythmia, etc.), medication use, and history of stroke (*p* > 0.05) (Table 1).

**Table 1:** Comparison of sociodemographic and clinical characteristics of treatment groups.

Variable	Ablation mean ± SD (min–max)	Cardioversion mean ± SD (min–max)	*p*
Age	59.9 ± 13.4 (30.0–79.0)	61.2 ± 8.9 (32.0–77.0)	0.668*
Body mass index (BMI)	27.6 ± 4.8 (19.1–36.4)	25.5 ± 4.5 (19.1–35.9)	0.101*

**Variable**	**Level**	**Ablation**		**Cardioversion**		***p***
		***n***	**%**	***n***	**%**	
Gender	Female	8	29.6	13	43.3	0.426**
	Male	19	70.4	17	56.7	
Marital status	Married	18	66.7	23	76.7	0.587**
	Single	9	33.3	7	23.3	
Education	Literate/primary school	7	25.9	7	23.3	0.769***
	Secondary school	8	29.6	8	26.7	
	High school	7	25.9	12	40	
	Associate degree—Bachelor's degree	4	14.8	3	10	
	Master's degree and above	1	3.7	0	0	
Employment status	Employed	6	22.2	8	26.7	0.935**
	Unemployed—Retired	21	77.8	22	73.3	
Smoking	Yes	11	40.7	12	40	1**
	No	16	59.3	18	60	
Presence of chronic disease	Yes	22	81.5	27	90	0.457***
	No	5	18.5	3	10	
Hypertension (HT)	Yes	17	63	16	53.3	0.641**
	Hayır	10	37	14	46.7	
Diabetes mellitus (DM)	Yes	6	22.2	8	26.7	0.935**
	Hayır	21	77.8	22	73.3	
Chronic obstructive pulmonary disease (COPD)	Yes	2	7.4	3	10	1***
	Hayır	25	92.6	27	90	
Asthma	Yes	1	3.7	1	3.3	1***
	Hayır	26	96.3	29	96.7	
Heart failure (HF)	Yes	3	11.1	9	30	0.109***
	No	24	88.9	21	70	
Arrhythmia	Yes	27	100	30	100	1***
Medication use	Yes	26	96.3	30	100	0.474***
	No	1	3.7	0	0	
History of stroke	Yes	4	14.8	3	10	0.697***
	No	23	85.2	27	90	

*Note:* Student's *t*‐tests *p* < 0.05; (*) Pearson's chi‐square tests *p* < 0.05 (**).

Fisher's exact test *p* < 0.05 (***).

During the 30–45 day follow‐up period, no major adverse events were reported. A limited number of patients experienced recurrence of AF symptoms; however, recurrence rates did not significantly differ between the cardioversion and ablation groups.

In both treatment groups, statistically significant changes were observed in quality of life (AFEQT), anxiety (BAI), and sleep quality (PSQI) scores both before and after treatment (Table 2).

**Table 2:** Comparison of pre‐ and post‐procedural AFEQT, BAI, and PSQI scores in the cardioversion and ablation groups.

Comparasion	Criterion	*p* value	Adjusted *p* value
Before and after ablation	AFEQT	0.000015*	0.000045
	BAI	0.007196*	0.0144
	PSQI	0.091957*	0.092
Before and after cardioversion	AFEQT	0*	< 0.0001
	BAI	0*	< 0.0001
	PSQI	0.000004*	0.000004
Between groups (post)	AFEQT	0.176361**	0.18
	BAI	0.000303**	0.00091
	PSQI	0.011321**	0.0226

*Note: p* values for all multiple comparisons across the three scales were corrected by the Holm method.

* Paired two‐sample *t*‐test (within‐group pre‐ vs. post‐treatment comparisons).

** Welch's unequal‐variance *t*‐test (post‐treatment scores compared between ablation and cardioversion groups).

In the ablation group, there was a notable increase in quality of life (AFEQT: mean +21.19; adjusted *p* = 0.000045) and a significant reduction in anxiety levels (BAI: mean –4.70; *p* = 0.0144). However, the improvement in sleep quality (PSQI: mean –0.59) was not statistically significant (*p* = 0.092).

In the cardioversion group, a significant improvement in AFEQT scores (mean +10.86; *p* < 0.0001), a significant decrease in anxiety levels (BAI: mean −8.63; *p* < 0.0001), and a significant improvement in sleep quality (PSQI: mean −1.10; *p* = 0.000004) were observed after intervention compared to before intervention.

When comparing the two treatment groups, no significant difference was found in post‐treatment AFEQT scores (*p* = 0.18). However, the cardioversion group showed significantly more favorable outcomes in anxiety (BAI, *p* = 0.0009) and sleep quality (PSQI, *p* = 0.0226) compared to the ablation group (Table 2).

Linear regression analyses revealed significant and strong correlations between quality of life, anxiety, and sleep quality both before and after treatment.

**Figure 1:**
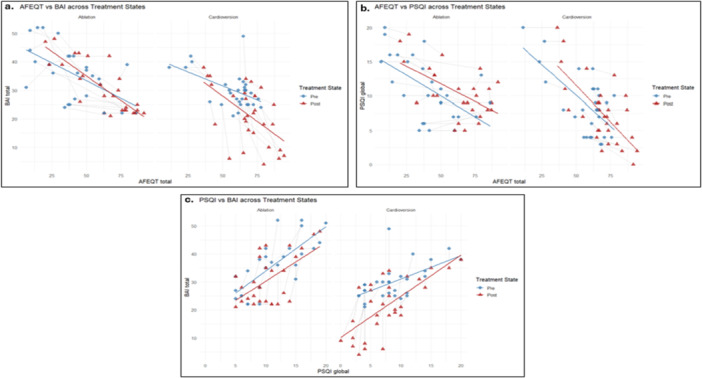
Linear regression analyses demonstrating the relationships between quality of life (AFEQT), anxiety (BAI), and sleep quality (PSQI) before and after cardioversion or catheter ablation. (a) AFEQT vs. BAI across Treatment State, (b) AFEOT vs. PSQI across Treatment State, and (c) PSQI vs. BAI across Treatment State.

In both the ablation (*n* = 27) and cardioversion (*n* = 30) groups, a higher quality of life was strongly associated with lower anxiety levels (Figure 1a). In the ablation group, the pre‐treatment regression slope was –0.247 (SE = 0.071, *p* = 0.0019) and increased to –0.342 (SE = 0.049, *p* = 2.7 × 10^−7^) post‐treatment, indicating that each 1‐point increase in AFEQT score corresponded to a 0.34‐point decrease in BAI score. A similar pattern was observed in the cardioversion group, with the slope improving from –0.195 (SE = 0.063, *p* = 0.0045) to –0.355 (SE = 0.099, *p* = 0.0012).

A positive correlation between quality of life and sleep quality was also maintained (Figure 1b). In the ablation group, the pre‐treatment slope was –0.121 (SE = 0.034, *p* = 0.0015), and post‐treatment it was –0.103 (SE = 0.029, *p* = 0.0015). In the cardioversion group, the slopes were –0.179 (SE = 0.038, *p* = 7.2 × 10^−5^) pre‐treatment and –0.211 (SE = 0.040, p = 1.4 × 10^−5^) post‐treatment. These results indicate that a better quality of life was associated with improved sleep quality.

Lastly, a negative relationship was observed between anxiety and sleep quality (Figure 1c). In the ablation group, the slope decreased from 0.366 (SE = 0.063, *p* = 4.5 × 10^−6^) pre‐treatment to 0.242 (SE = 0.069, *p* = 0.0016) post‐treatment. In the cardioversion group, the slope changed from 0.407 (SE = 0.107, *p* = 7.3 × 10^−4^) to 0.331 (SE = 0.064, *p* = 1.8 × 10^−5^).

Descriptive statistics of AFEQT, BAI, and PSQI scores before and after treatment for both groups are presented in Table 3. Overall, the cardioversion group demonstrated more consistent and pronounced improvements.

**Table 3:** Descriptive statistics of pre‐ and post‐treatment survey results.

Grup	AFEQT (mean ± SD)	95% Confidence interval	BAI (mean ± SD)	95% Confidence interval	PSQI (mean ± SD)	95% Confidence interval
Before ablation	40.33 ± 21.75	31.72−48.93	35.81 ± 9.42	32.08−39.54	11.19 ± 4.54	9.38−12.98
After ablation	61.52 ± 21.04	53.19−69.84	31.11 ± 8.87	27.60−34.62	10.59 ± 3.72	9.11−12.06
Before cardioversion	57.50 ± 16.13	51.47−63.52	30.07 ± 6.25	27.73−32.40	8.73 ± 4.37	7.10−10.36
After cardioversion	68.36 ± 15.92	62.41−74.31	21.43 ± 10.06	17.67−25.19	7.63 ± 4.77	5.85−9.41

*Note:* Standard error (SE) and 95% confidence intervals (CI) for each group were calculated using the standard formula of the Gaussian normal distribution: CI = mean ± 1.96 × SE.

In the ablation group, the mean AFEQT score increased from 40.33 (CI: 31.72–48.94) to 61.52 (CI: 53.20–69.85), indicating a significant improvement in quality of life. BAI scores showed a modest decline from 35.81 to 31.11, and PSQI scores slightly decreased from 11.19 to 10.59, suggesting limited improvement in sleep quality.

In the cardioversion group, changes were more substantial across all measures. The AFEQT score increased from 57.50 (CI: 51.48–63.52) to 68.36 (CI: 62.42–74.31), indicating improved functional status. BAI scores decreased from 30.07 to 21.43, and PSQI scores dropped from 8.73 to 7.63, showing a meaningful enhancement in sleep quality.

In summary, although AFEQT scores changed more favorably after the application of her two interventions, the cardioversion groups experienced greater changes in anxiety and sleep disturbance scores compared to the ablation group.

**Figure 2:**
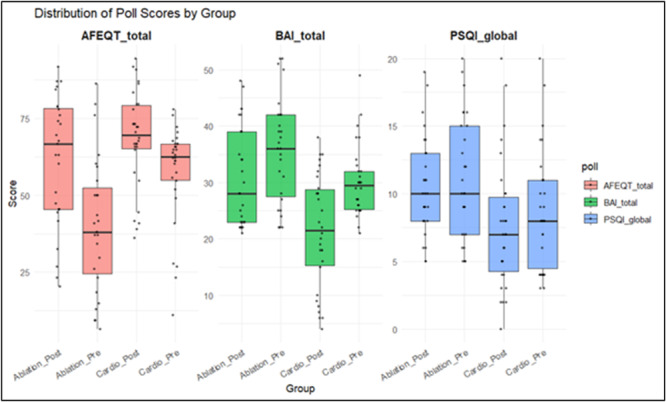
Boxplot of distribution statistics of polls across the treatment types and states.

Figure 2 and Table 3, which compare median and variance distributions across surveys, support these findings. AFEQT scores improved significantly post‐treatment in both the ablation (mean +21 points) and cardioversion (mean +11 points) groups. BAI and PSQI scores also decreased. Significant results were observed in this unit and clinically in all measurement units after the intervention compared to before the intervention.

## 4 Discussion

This study evaluated changes in quality of life, anxiety levels, and sleep quality in patients diagnosed with AF who underwent elective catheter ablation or electrical cardioversion. The findings demonstrated that improvements in quality of life and psychological well‐being were observed following both interventions; however, greater reductions in anxiety and sleep disturbance scores were observed in the cardioversion group.

The significant improvements in quality of life after both ablation and cardioversion procedures are consistent with previous studies. Recent studies have demonstrated that catheter ablation in patients with AF leads to significant improvements in symptom burden and quality of life [18, 19]. In a large multicenter randomized trial, Mark et al. (2019) found that catheter ablation was clinically and statistically superior to medical therapy in terms of symptomatic improvement and quality of life among AF patients. Similarly, Savelieva and Camm [20] emphasized that cardioversion effectively improved symptom control and quality of life in the short term. In the present study, a 21‐point increase in AFEQT scores was observed in the ablation group, which was statistically significant. Although the cardioversion group also showed a marked increase in quality of life, no significant difference was found between the two groups post‐treatment. This suggests that both interventions offer comparable improvements in quality of life, with cardioversion potentially delivering benefits more rapidly.

A particularly noteworthy finding of this study was the significant reduction in anxiety levels across both groups. The decline in BAI scores was more pronounced in the cardioversion group, aligning with existing literature. Zhang et al. [21] emphasized that symptom control in AF patients contributes to psychological relief, while Lee et al. [22] suggested that rapid improvements in anxiety following cardioversion may be linked to increased confidence in rhythm control.

The results regarding sleep quality represent a unique contribution of this study. The cardioversion group experienced a statistically significant improvement in PSQI scores, while the ablation group showed a nonsignificant change. Similarly, Wood et al. (2022) reported that although sleep quality improved 1 month after ablation, the change was not statistically significant. The difference in sleep quality outcomes between groups may be due to the faster rhythm stabilization achieved through cardioversion. Literature has established that AF adversely affects sleep, and rhythm control can improve sleep quality [23, 24].

Furthermore, our findings revealed strong and consistent correlations between a higher quality of life and lower anxiety levels, as well as better sleep quality. These relationships are well‐supported in the literature [25, 26] and further emphasize the need for a comprehensive, multidimensional approach to AF treatment.

In conclusion, this study demonstrates that both cardioversion and ablation have positive effects not only on physiological outcomes but also on psychological and lifestyle‐related parameters in AF patients. The rapid psychological benefits observed after cardioversion may contribute to its preference in patient‐centered treatment planning. However, ablation has been shown in other studies to offer superior long‐term rhythm control [27]. Therefore, treatment decisions should be made through a multidisciplinary approach that considers individual patient needs, clinical characteristics, and psychosocial status.

### 4.1 Limitations

This study was conducted at a single center with a limited number of participants, which restricts the generalizability of the findings. Data were collected through self‐report questionnaires, which may introduce subjectivity and response bias. Additionally, the follow‐up period was limited to approximately 30–45 days after the procedures; therefore, the findings reflect only short‐term changes in quality of life, anxiety, and sleep quality. Long‐term sustainability of these observed changes, particularly in relation to AF recurrence and rhythm maintenance, could not be evaluated.

## 5 Conclusion

This study compared pre‐ and postoperative results in patients with AF who underwent both catheter ablation and electrical cardioversion procedures. Both interventions resulted in positive changes in quality of life, anxiety levels, and sleep quality. However, patients who underwent cardioversion showed more significant reductions in anxiety levels and sleep disturbance scores. These findings highlight the importance of evaluating psychosocial and lifestyle outcomes in addition to rhythm control strategies in patients with AF. Considering patient‐reported outcomes such as anxiety, sleep quality, and quality of life can contribute to more comprehensive and patient‐centered treatment planning. Since the current study evaluated short‐term outcomes in a single‐center cohort, multicenter prospective studies with longer follow‐up periods are needed to better elucidate the long‐term clinical and psychosocial impacts of these interventions.

## Author Contributions

**Besey Oren:** study conception, designed data analysis, approved the final version to be published, wrote the first draft of the manuscript, and approved the final version to be published. **Busra Zehra Buyukkilic:** study conception, designed data analysis, approved the final version to be published. **Kubra Soydas:** study conception, designed data analysis, wrote the first draft of the manuscript, and approved the final version to be published. **Ekin Oren:** designed data analysis, wrote the first draft of the manuscript, and approved the final version to be published.

## Ethics Statement

Written approval (decision number: 20/17, dated: 01.12.2023) was received from the Scientific Research Ethics Committee of Health Sciences University to implement the study. Institutional permission was obtained for data collection. All participants were informed about the research, and their informed consent was received.

## Conflicts of Interest

The authors declare no conflicts of interest.

## Data Availability

The data that support the findings of this study are available from the corresponding author upon reasonable request.
